# Dye-Doped Fluorescent Silica Nanoparticles for Live Cell and *In Vivo* Bioimaging

**DOI:** 10.3390/nano6050081

**Published:** 2016-04-27

**Authors:** Wen-Han Zhang, Xiao-Xiao Hu, Xiao-Bing Zhang

**Affiliations:** Molecular Sciences and Biomedicine Laboratory, State Key Laboratory for Chemo/Biosensing and Chemometrics, College of Chemistry and Chemical Engineering, College of Biology and Collaborative Innovation Center for Chemistry and Molecular Medicine, Hunan University, Changsha 410082, China; yibenwuyue@163.com

**Keywords:** silica nanoparticle, organic fluorescent dye, bioimaging, *in vivo*

## Abstract

The need for novel design strategies for fluorescent nanomaterials to improve our understanding of biological activities at the molecular level is increasing rapidly. Dye-doped fluorescent silica nanoparticles (SiNPs) emerge with great potential for developing fluorescence imaging techniques as a novel and ideal platform for the monitoring of living cells and the whole body. Organic dye-containing fluorescent SiNPs exhibit many advantages: they have excellent biocompatibility, are non-toxic, highly hydrophilic, optically transparent, size-tunable and easily modified with various biomolecules. The outer silica shell matrix protects fluorophores from outside chemical reaction factors and provides a hydrophilic shell for the insoluble nanoparticles, which enhances the photo-stability and biocompatibility of the organic fluorescent dyes. Here, we give a summary of the synthesis, characteristics and applications of fluorescent SiNPs for non-invasive fluorescence bioimaging in live cells and *in vivo*. Additionally, the challenges and perspectives of SiNPs are also discussed. We prospect that the further development of these nanoparticles will lead to an exciting breakthrough in the understanding of biological processes.

## 1. Introduction

Living cells and the human body are very complex systems because of the high diversity in organic composition and distinct temporal-spatial variation in metabolism. To properly understand life activities, we need to monitor the individual chemical interactions happening *in vivo*. Recently, *in vivo* imaging technologies have attracted more attention for being effective to reveal how activities occur in the physiological state [1].

Common traditional organic fluorescent dyes, such as, Cy3, fluorescein, 5-carboxyfluorescein (FAM), Cy5, carboxytetramethylrhodamine (TRAMA), fluorescein isothiocyanate (FITC) and Rhodamine B, are widely used reagents in bioimaging applications. However, there are still some defects, such as low fluorescence intensity, low photostability and insufficient stability, which make the traditional organic fluorophores not suitable for high-sensitivity detection and real-time monitoring. Increasingly studied in bioimaging, nanomaterials are a promising application prospect *in vivo* [2,3,4,5]. Fluorescent nanomaterials overcome some limitations of organic fluorescent dyes, such as poor hydrophilicity, rapid photobleaching and low quantum yield [6,7]. Among all kinds of fluorescent nanomaterials developed for bioanalysis, nanoparticles are always the core of various nanosystems used for bioimaging *in vivo* [8]. Moreover, nanoparticles with a modified surface can bind to their targets specifically in an individual organism environment and convert interactions into readable signals [6]. Because their targets usually exist at the nanoscale, the nanoparticles with nanometer scale can bring as few physical perturbations as possible to the vulnerable live cells and the entire body [9]. Furthermore, nanoparticles have the ability to conjugate to several ligand molecules, which makes it very easy to design probes and to optimize the labeling ratio for achieving higher sensitivity in the complex detection environment. In the past decade, highly ﬂuorescent quantum dots (QDs), one category of nanoparticles with an ultra-small size, have been used as an intriguing and novel class of fluorescent sensor platform due to their outstanding optical characteristics, such as no photobleaching, tunable emission light from the visible region to the near-infrared (NIR) region and controllable particle size [10]. However, the disadvantages, such as poor solubility, toxicity and blinking, confine the direct applications in live cells and the entire body [11].

After overcoming the above difficulties, we expect to achieve these as follows: small size to enter cells for bioimaging, high sensitivity for effective detection, fast response to monitor the life metabolism of targets, high abundancy, non-toxicity, good dispersibility in the biological environment, resistant to metabolic disintegration and photobleaching, high selectivity in the extremely complex organism [12,13,14].

With these properties, such as the excellent biocompatibility, non-toxicity, high hydrophily, optical transparency, tunable size and ease to covalently attach multifarious biomolecules (e.g., DNA, peptides, antibodies, RNA, proteins, *etc.*) to their surface, different fluorescent silica nanoparticles (SiNPs) have been widely used as an extremely significant platform to acquire more applicable bioimaging probes in living cells and the entire body [15,16,17,18,19,20,21,22]. Different from using organic fluorescent dyes alone, dye-doped SiNPs that contain thousands of dye molecules demonstrate the superiority of the highly amplified optical signal [23]. In addition, SiNPs’ encapsulation can greatly reduce exterior quenching and degrading, while enhancing the chemical stability and biocompatibility of the fluorescent dyes, which can further improve dye photo-stability and meet the need of real-time and long-time tracking *in vivo* [24]. For the bioimaging, monitoring and detecting research fields, functionalized fluorescent SiNPs became a more suitable choice due to their superior properties.

We will describe the recent research progress on the developments of functionalized fluorescent SiNPs in bioimaging applications in those aspects as follows. Firstly, the properties of the SiNPs and common synthetic methods will be generally summarized. Next, recent progress about functionalized SiNPs with organic fluorescent dye and inorganic nanoparticles in bioimaging will be described. Finally, the challenges and perspectives of functionalized fluorescent SiNPs will be discussed.

## 2. Properties and Synthetic Method of SiNPs

### 2.1. Properties of SiNPs

The functionalized fluorescent SiNPs are the silica matrix combined with traditional organic fluorophores and silica-coated fluorescent nanoparticles. The advantages of the spherical SiNPs include:

1.  SiNPs are extremely hydrophilic and convenient to prepare, synthesize and separate.
2.  Silica material is biocompatible and non-toxic and, thus, causes little damage to living cells.
3.  Silica material can let excitation light and emission pass through without changing their characteristics.
4.  SiNPs attach biomolecules effectively and can be easily modified with a variety of functional groups, such as poly(ethylene glycol) (PEG), hydroxyl (OH), carboxyl (COOH), phosphonate (CH_3_HPO_2_) and amine (NH_2_) on their surface.
5.  The silica matrix can encapsulate dyes and fluorescent nanoparticles without changing the optical character in convenient methods [25], and the stability of the dye can be improved after being doped in silica matrix.
6.  The controllable particle size of SiNPs makes them suitable to apply for *in vivo* bioimaging.

### 2.2. Synthetic Method of SiNPs

**Figure 1:**
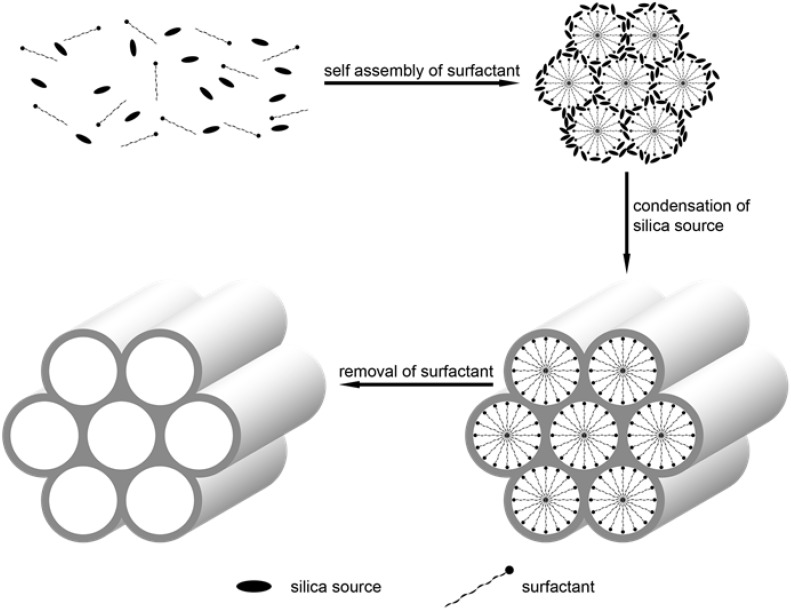
Simple procedure synthesis of mesoporous silica nanoparticles.

The common synthetic methods of SiNPs are the reverse micelle method and the Stöber method. Reverse microemulsion, one class of microemulsions, consists of a homogeneous transparent or translucent mixture of oil, water, an assistant of the surfactant and surfactant molecules that are isotropic and thermodynamically stable [26,27,28]. Since the electrostatic attraction between dyes and the silica shell would be predominant for the formation of stable dye-containing SiNPs, polar and hydrophilic fluorescent dyes have been easily entrapped into SiNPs with this synthetic method [20,24,29]. Various kinds of fluorophores were encapsulated simultaneously in one SiNP in a similar way [30,31]. The Stöber method is another commonly-used method, founded by Werner Stöber *et al.* in 1968, for synthesizing monodisperse silica [32]. In the method, the SiNPs are synthesized by adding siloxane precursors, such as tetraethyl orthosilicate (TEOS), into ethanol and ammonium hydroxide in a certain percentage. With the covalent attachment, a variety of fluorophores have been incorporated into the SiNPs by this method. In addition, other nanomaterials can be encapsulated into one SiNP in this way, such as QDs and magnetic nanoparticles [33,34]. The nanocomposites also have multiple features that make them versatile in bioimaging. Silica nanoparticles with a honeycomb-like pore structure, large pore volume and uniform pore size were known as mesoporous silica nanoparticles (MSNs) [35]. MSNs were firstly synthesized by using surfactants as structure-directing agents in the early 1990s [36,37]. One of the most widely-used types of MSNs, was synthesized and named as MCM-41 [36]. MCM-41 was synthesized with the surfactant of cetyltrimethylammonium bromide (CTAB), tetraethyl orthosilicate (TEOS) or sodium metasilicate (Na_2_SiO_3_) and alkali as the catalyst. After removing the surfactant, the uniform pore structure can be formed. The synthesis procedure of MSN is shown as Figure 1. MSNs have become more attractive in biomedical and bioimaging for these characteristics [38,39,40].

## 3. Bioimaging

Due to the properties of the high efficiency, sensitivity and selectivity, organic fluorescent dyes have been widely used in bioimaging applications [41,42,43,44]. However, organic fluorescent dyes have many disadvantages, especially rapid photobleaching. The photobleaching of the dyes is a result of the many molecules in cellular matrix capable of inducing photochemical reactions. One solution is to encapsulate the fluorescent dyes, which protects them from chemical reaction, and thus, the chemical stability and biocompatibility of the fluorescent dyes can be greatly enhanced. Many kinds of encapsulation approaches have been proposed, including using lipid micelles, polymer matrices or silica matrix [20,45,46,47,48]. Silica matrix became a more attractive choice due to those characteristics, such as biocompatibility, non-toxicity, water-solubility, size control and easy modification.

### 3.1. Traditional Fluorescent Molecular

Traditional fluorescent dye-doped SiNPs are used in the bioimaging field for the unique photo-stability, biocompatibility and easy modification of their surface. For example, Van Blaaderen *et al.* have successfully incorporated FITC into the silica matrix [25]. Tan and co-workers optimized the preparation process of dye-doped SiNPs [49,50]. Recently, in order to enhance the fluorescence of dye-doped SiNPs, a simple method was based on metal-enhanced fluorescence (MEF) for aggregating amounts of gold nanoparticles(GNPs) around Rhodamine B-doped SiNPs [51]. MEF is an effective tool to increase the photo-stability and brightness of the fluorophore at the same time at an appropriate distance between the nanoparticle and the fluorescent dye molecules (~5 to 30 nm) [52,53]. Firstly, they used a modified Stöber method and a core-shell design to produce the two types of dye-doped SiNPs. Next, they used dithiocarbamate chemistry to immobilize GNPs on the amine-coated SiNPs in water/ethanol solutions. Finally, after being optimized, the fluorescence brightness of the SiNPs achieved an enhancement of over 200-fold successfully.

**Figure 2:**
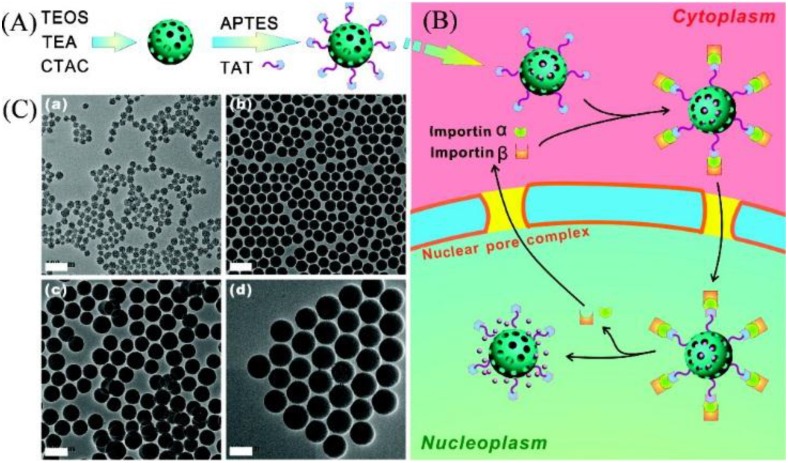
(**A**) Procedures for preparation amine group- and TAT peptide-conjugated mesoporous silica nanoparticles (MSNs); (**B**) an illustration of doxorubicin (Dox)@MSNs-TAT for targeting nuclei of cancer cells and delivering/releasing drugs directly into nuclei; (**C**) Transmission electron microscope transmission electron microscope (TEM) images of MSNs with different sizes. Scale bars: 100 nm. Reproduced with permission from [55]. Copyright 2012 American Chemical Society.

Recently, an aptamer-modified SiNPs system has also been reported [54]. It is shown for the first time that functionalized fluorescent SiNPs modified with two types of aptamers can target biomarkers of breast cancer. After being synthesized by the common reverse microemulsion method, the fluorescent SiNPs were modified with PEG. Thereafter, an immobilization of avidin onto the PEG-coated SiNPs as prepared. The two biotin-aptamers were connected with the avi-SiNPs via biotin-avidin interactions. This novel diagnostic system could be an excellent platform for an extensive diagnosis of breast cancer. They cultured four different breast cancer cell lines with overexpressed biomarkers to demonstrate the binding affinity of the functionalized SiNPs and the excellent high binding appearance of the dual-SiNPs to all of those cell lines. MSNs, one special category of SiNPs, with a uniform pore size and large pore volume, are anticipated to be extra ordinary nanomaterials for bioimaging and drug delivery. For instance, Shi’s group reported that TAT peptide-conjugated MSNs can be used for nuclear-targeted drug delivery [55]. They immobilized FITC on the inner walls of the pores to trace the nanoparticles and conjugated TAT peptide onto MSNs (Figure 1). TAT peptide, one of the nuclear localization signals (NLS), is an amino acid sequence that transports protein into the cell nucleus by binding import receptors importin α and importin β [56,57]. In this way, heterogeneous material, such as nanoparticles with an appropriate size, can target the nuclear pore complexes of the cancer cells and enter the nucleus [58]. Moreover, to take advantage of the high payload of the MSNs, doxorubicin (Dox), one of the most widely-used anticancer drugs, was loaded for further treatment. The multiple SiNPs displayed excellent effects on bioimaging and therapeutics. The size of the MSN-TAT has a very important influence on passing nuclear pore complexes. As we all know, most nuclear pore complexes have a diameter of 20−70 nm. The size of nanoparticle has an influence on the delivery efficiency [59]. To find out the suitable size for the nuclear internalization, four MSNs with different sizes (25 nm, 50 nm, 67 nm, 105 nm) have been prepared. The size of MSNs is small enough to ensure the entering through the nuclear pore. However, MSNs with a small size (<50 nm) are quite hard to synthesize. In this paper, the particle size of MSNs was obtained as 25 nm (triethanolamine (TEA), 0.08 g), 50 nm (TEA，0.06 g) *etc.*, via changing the amount of TEA used under the same conditions*.* The greater the amount of TEA used, the smaller the size. The cellular confocal laser scanning microscopy (CLSM) of MSNs-TAT with different sizes for 4-, 8- and 24-h incubations is shown in Figure 2. It turned out that 25−50-nm MSNs conjugated with TAT peptide are the most appropriate for transmembrane transport.

**Figure 3:**
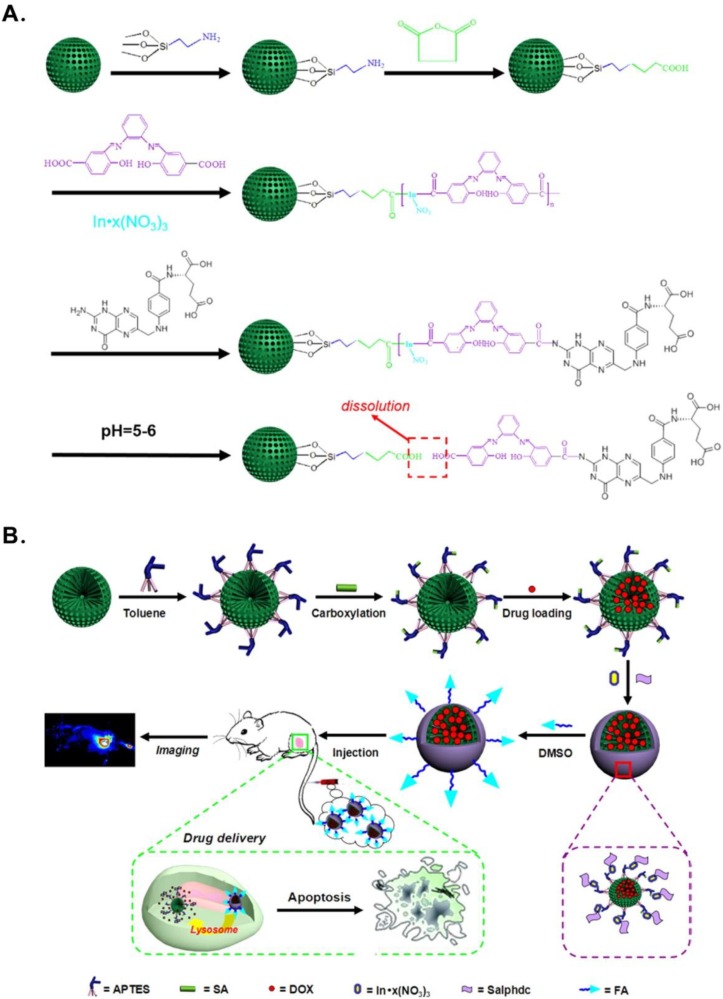
(**A**) Chemical reaction routes of pH-responsive hierarchical poreSiNP (HPSN)-*N*,*N*-phenylenebis(salicylideneimine)dicarboxylic acid (Salphdc)-folate (FA) nanosystem; (**B**) the tumor therapy and bioimaging *in vivo* of the drug-loaded HPSN-Salphdc-FA nanosystem. Reproduced with permission from [39]. Copyright 2015 American Chemical Society.

**Figure 4:**
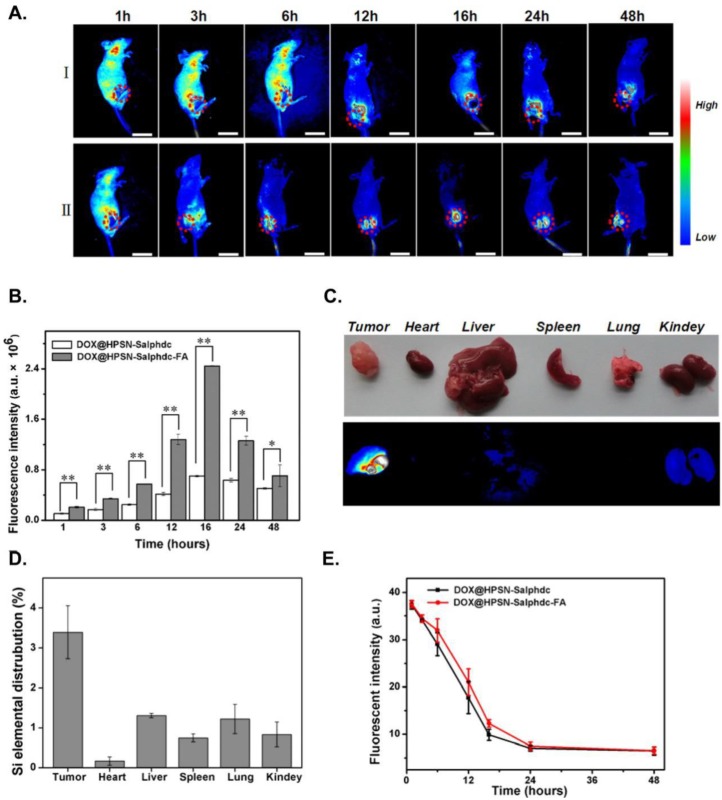
(**A**) Whole-body real-time fluorescence imaging of DOX@HPSN-Salphdc (I) and DOX@HPSN-Salphdc-FA (II) at different hours. Scale bars: 3 cm. (**B**) Histogram of the fluorescence intensity of tumors issue treated with Dox@HPSN-Salphdc and Dox@HPSN-Salphdc-FA at each interval, respectively. (**C**) Images of mainly organs and (**D**) quantitative energy dispersive spectrometry analysis after injection of DOX@HPSN-Salphdc-FA for 16 h. (**E**) The fluorescence intensities of both nanoparticles in blood over time. The error bars indicate the mean ± SD (*n* = 4). * *p* < 0.05 and ** *p*< 0.01. Reproduced with permission from [39]. Copyright 2015 American Chemical Society.

Recently, folate (FA)-conjugated pH-responsive SiNPs for targeted drug delivery and bioimaging have also been used by Cai’s group [39]. The design of the controlled drug-release nanoplatform was shown as (Figure 3). They chose SiNPs with hierarchical pores (HPSNs) as the nanocontainers to acquire a better drug loading than MSN for moderately decreasing pore sizes from the surface of particles to the center. Next, the HPSNs were modified by amino and carboxyl groups for a further conjugation with *N*,*N*-phenylenebis(salicylideneimine)dicarboxylic acid (Salphdc). Salphdc, a molecule with a unique autofluorescence characteristic and good biocompatibility, was used as a fluorescence imaging agent and gate-keeper for the metal-organic framework thin films interacting with metal ions [60,61]. Moreover, the bond between HPSNs and Salphdc was sensitive to acid, which made the HPSN complex be pH-responsive. Finally, FA is grafted to the HPSN with a Salphdc shell as a common targeting segment. Generally, many human tumors overexpress FA receptors, while healthy cell shave no FA receptor. The recognition between the receptor and ligand makes FA a commonly-used desirable target motif [62]. To make sure that the Salphdc-capped HPSN nanoparticle could be applied for bioimaging *in vivo*, both Dox@HPSN-Salphdc and Dox@HPSN-Salphdc-FA were injected into tumor-bearing mice. The whole-body real-time fluorescence imaging at different times, the fluorescence intensity of tumor after injection at each interval, the nanoparticle distribution in tissues and the fluorescence intensities of both nanoparticles in blood over time have been studied (Figure 4).

Beside the single imaging modality, Piao *et al.* recently synthesized multimodal imaging core-shell nanoparticles for the precision of tumor diagnosis using a single and simple injection of contrast agent [63]. The core-shell nanoparticles consist of iron oxide nanoparticle as the core and fluorescent silica as the shell. With these characteristics, the core-shell SiNPs have the ability to image the tumor with fluorescence and magnetic resonance imaging (MRI). MRI is a mature clinical imaging technique for non-invasive disease diagnosis or post-therapy evaluation for diseases. The sensitivity of MRI can be extraordinarily enhanced with positive (T1-weighted, bright) or negative (T2-weighted, dark) contrast agents [64]. To prove the tumor-selective delivery of fluorescent silica nanoshell-coated iron oxide nanoparticles via intravenous injection, *in vivo* fluorescence imaging of four different SKOV-3 tumor (one kind of human ovarian tumor)-bearing mouse models has been performed. They also showed *ex vivo* fluorescence imaging to identify the organ distribution of the core-shell nanoparticles in xenografts. Moreover, *in vivo* and *in vitro* MRI have also been performed.

### 3.2. Near-Infrared Fluorescent Dye

The signal of the conventional fluorescent dyes can be seriously affected by the autofluorescence from biological tissues, which always results in severe interference and limited penetration depth. To achieve a better imaging quality *in vivo*, methylene blue (MB), one of the most extensively-used and inexpensive NIR fluorescent dyes, was selected to be doped into the SiNPs to construct a novel NIR fluorescence imaging system [65]. Moreover, the imaging probe was very stable with a considerable NIR fluorescence intensity, and the dye leakage is very low. Jin *et al.* designed a multifunctional mesoporous SiNP platform for controlled drug release, targeted delivery and imaging *in vivo* [66]. In this design, FA was used as a cancer-targeting segment and Cy5 as an NIR fluorescent dye for bioimaging. Firstly, they prepared carboxyl-functionalized MSN(MSN/COOH) and coated MSN/COOH with NH_2_-PEG-FA. After being modified with Cy5 via the 1-Ethyl-3-(3-dimethylaminopropyl)carbodiimide hydrochloride(EDC)/*N*-Hydroxysulfosuccinmide sodium salt (Sulfo-NHS) coupling method, the system MSN/COOH-PEG-FA is available for labelling. The electrostatic attraction between the Dox and nanoparticles is the main factor for the Dox loading. Considering the functionalization with negatively-charged carboxyl, we can load a large amount of Dox into the mesoporous SiNPs. Moreover, it is also found that the release of Dox from the MSN/COOH-PEG-FA nanosystems was related to the medium pH. The electrostatic interactions between the Dox and the carboxyl were decreased, while the electrostatic repulsion between them enhanced from pH 7.4 to pH 5.0. As the pH is lower in tumor cells than in normal cells, the delivery efficiency of the MSN/COOH-PEG-FA nanoparticles enhanced drastically. The imaging in mouse tumor after the injection of MSN/COOH-Cy5 and free Cy5 for different hours has been also investigated. Based on these characteristics, mesoporous SiNPs have the ability for both effective imaging and treatment of diseases.

**Figure 5:**
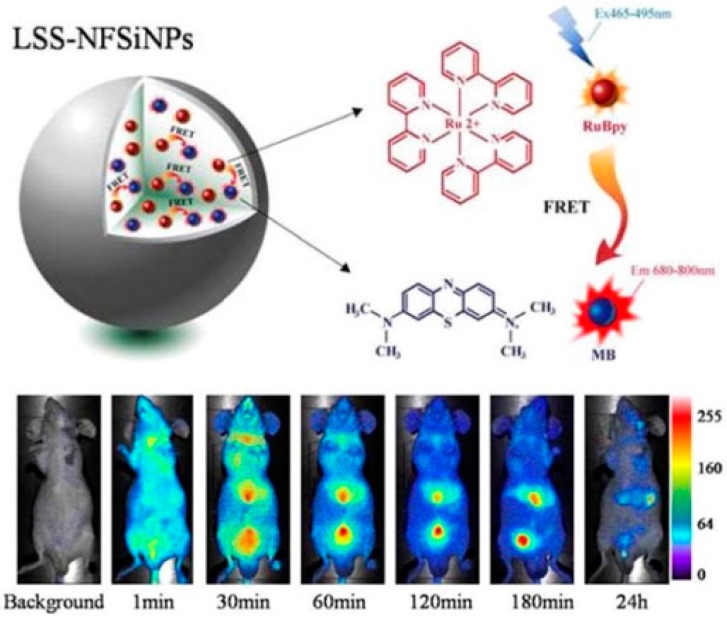
Experimental design for the large stokes shifting NIR fluorescent SiNPs (LSS-NFSiNPs) and real-time abdomen fluorescence resonance energy transfer (FRET) imaging of mice intravenously injected with the LSS-NFSiNPs. Reproduced with permission from [30]. Copyright 2012 American Chemical Society.

To solve the problem of background and interference between the emitting signals and the excitation light, Wang and co-workers presented a novel and ideal fluorescence resonance energy transfer (FRET)-mediated large Stokes shifting NIR fluorescent SiNPs (LSS-NFSiNPs) [30]. FRET is an interaction between a donor molecule and an acceptor molecule, which is very sensitive to the distance between the fluorophores [67,68]. The water-soluble dyes of RuBpy and MB were chosen as a donor-acceptor pair for modal FRET and co-encapsulated inside a SiNP. The effectiveness of the LSS-NF SiNPs for depth-tissue fluorescent imaging in living animals has also been demonstrated (Figure 5). The large Stokes shift (>200 nm) and strong fluorescence signal of the NIR fluorescent SiNPs, which were caused by the energy transfer from RuBpy to MB occurring in the silica nanoparticles, have shown a promising future for medical imaging application.

A multimodal imaging SiNP probe labeled with NIR fluorescent dye has also been reported [69]. Firstly, they synthesized Cy5-conjugatedironoxide@silica core-shell nanoparticles. Due to the ability of the iron oxide to quench fluorescent materials [70,71], they synthesized Cy5-conjugated core@shell with different diameters to figure out the relationship between the distance and fluorescence quenching. Then, in the presence of hydrogen fluoride (HF), the silica shell is decomposed, and free dyes are released from the surface of the nanoparticles, which made the fluorescence of Cy5 dyes be fully recovered. They showed that the nanoparticles with a 113-nm diameter had the best fluorescence efficiency. In addition, the nanoparticles showed an effect on *in vivo* MRI and NIR fluorescence imaging.

**Figure 6:**
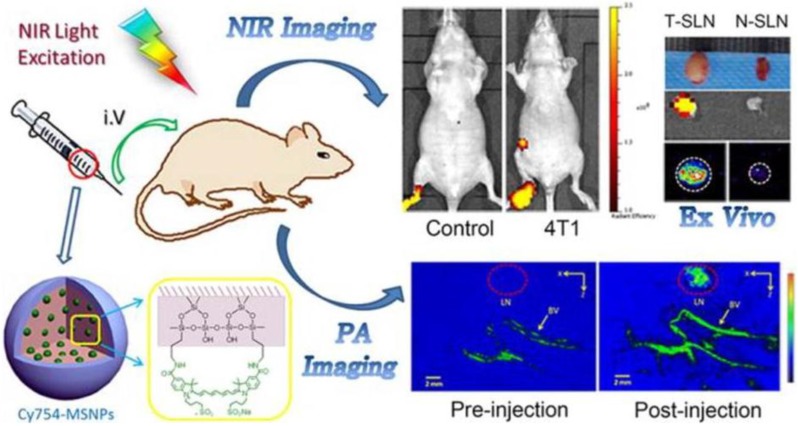
Schematic illustration of dye-loaded mesoporous SiNPs for both NIR fluorescent and photoacoustic (PA) imaging. Reproduced with permission from [72]. Copyright 2015 American Chemical Society.

Not only have MRI/NIR and PET/NIR dual-modality been used for *in vivo* imaging, but also a nanocontrast for photoacoustic (PA)/NIR imaging dual-modality has been applied in noninvasive imaging [72]. They developed an imaging nanocontrast based on NIR fluorescent dye-loaded mesoporous SiNPs for both NIR fluorescent and PA imaging (Figure 6). PA imaging is a biomedical imaging modality based on the photoacoustic effect. When the non-ionizing laser pulses are delivered into biological tissues, the absorbed energy will be converted into heat, leading to the transient of thermoelastic expansion and wideband ultrasonic emission. By changing the ratio of the dye and nanoparticles for simultaneous optimization of NIR and PA imaging, they found that mesoporous SiNPs loaded with 80 μM dyes generated both a moderate NIR emission and PA signals. *In vivo* fluorescence imaging and photoacoustic imaging are carried out in their study.

### 3.3. Two-Photon Fluorescent Dye

**Figure 7:**
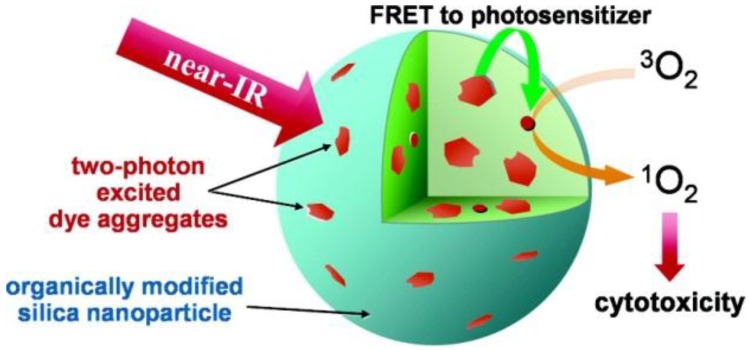
Schematic illustration of organically-modified two-photon photodynamic therapy SiNPs. Reproduced with permission from [74]. Copyright 2007 American Chemical Society.

Two-photon fluorescent dyes can be another choice to realize deep-tissue imaging, since they can prolong observation time, provide low fluorescence backnd, less light scattering and less tissue injury and better three-dimensional spatial localization [73]. Two-photon fluorescent dyes with a silica shell were synthesized, as well [73,74]. For example, nanoparticles consisted of a two-photon fluorescent dye and a photodynamic therapy (PDT) drug [74]. PDT, a light-activated novel therapeutic tool for curing cancer and other diseases, is triggered by photoexcitation of the photosensitizer to produce reactive oxygen to kill cells, such as peroxides, free radicals or singlet oxygen (^1^O_2_) [75]. With this feature, it brings therapeutics very precisely to the target, without damaging adjacent normal tissues. Two-photon fluorescent aggregates were used as an energy up-converting donor and a photosensitizing drug as an acceptor (Figure 7). Through the FRET process, the energy of near-IR light can efficiently transfer to the PDT drugs that were co-encapsulated in the SiNPs. In this way, the efficiency of two-photon photodynamic therapy can be remarkably enhanced.

**Figure 8:**
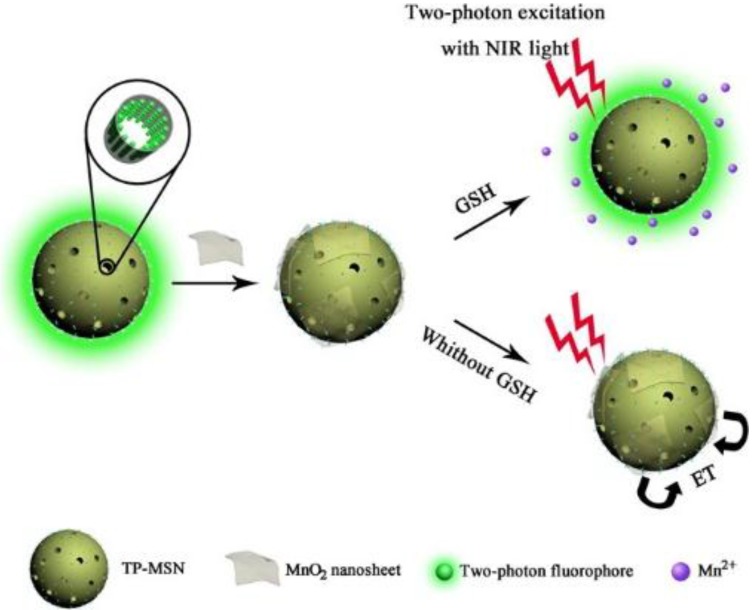
Schematic illustration of two-photon (TP)-MSNs@MnO_2_for glutathione detection. Reproduced with permission from [73]. Copyright 2014 American Chemical Society.

**Figure 9:**
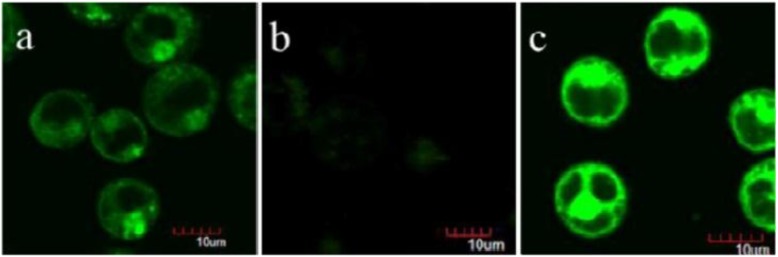
Two-photon confocal microscopy (CM) images of glutathione detection in living CEM cells. (**a**) CEM cells incubated with the TP-MSN@MnO_2_ nanoparticle; (**b**) CEM cells pretreated with glutathione scavenger and then incubated with the TP-MSN@MnO_2_ nanoparticle; (**c**) CEM cells pretreated with glutathione synthesis enhancer and incubated with the TP-MSN@MnO_2_ nanoparticle. Reproduced with permission from [73]. Copyright 2014 American Chemical Society.

In another application, Zhang and co-workers firstly reported a “turn-on” two-photon fluorescent dyes@MSN(TP-MSNs@MnO_2_) for the detection of glutathione in water solution and *in vivo* [73]*.* The imaging of two-photon fluorescence for the detection of glutathione in CEM cells (human T cell acute lymphoblastic leukemia cell line) and tissues has been achieved (Figure 8). They used MnO_2_nanosheets to quench the fluorescence of the two-photon dyes. Through electrostatic interaction between the negatively-charged quencher MnO_2_nanosheets and the positively-charged MSNs, the nanosheets were adsorbed onto the surface easily. If glutathione exists in the environment, MnO_2_ would be reduced into Mn^2+^, which leads the MnO_2_nanosheets to decompose, and consequently, a significant two-photon fluorescence nanosystem can be recovered. The confocal microscopy (CM) imaging experiment of glutathione in CEM cells demonstrated that the nanosensor can monitor the change of the glutathione in living cells (Figure 9). In addition, to take advantage of the deep-issue imaging of the two-photo fluorescent dye, the nanoplatform was investigated for imaging rat liver frozen slices as a model of tissue imaging for further application. It has clearly showed that the TP-MSNs@MnO_2_ nanoparticles are available for effective imaging of glutathione in living cells and at the tissue level with high sensitivity and tissue penetration.

### 3.4. Multiple Fluorescent Molecules

**Figure 10:**
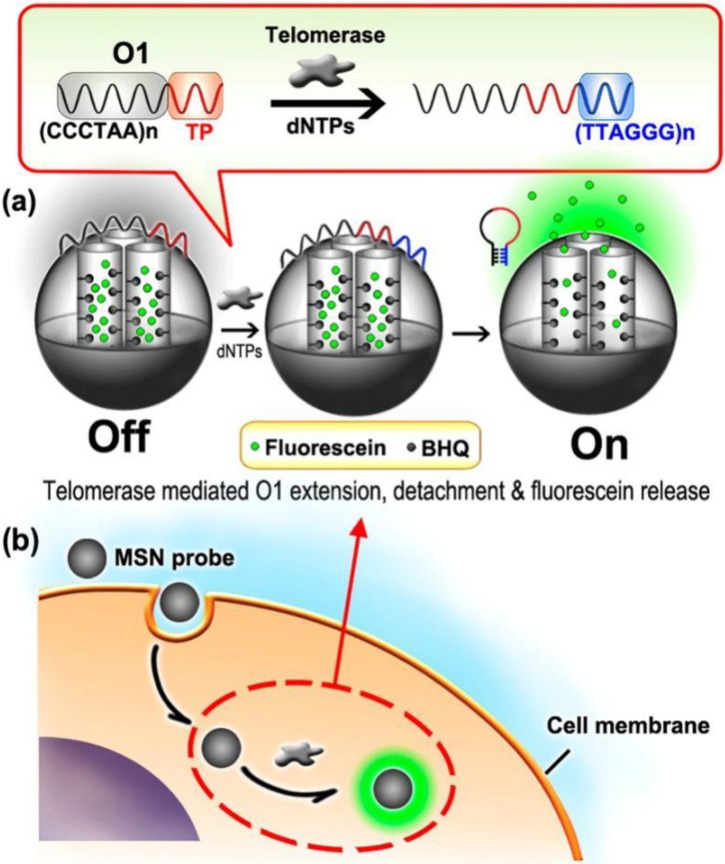
Schematic illustration of MSN probe-based intracellular detection of telomerase. Reproduced with permission from [76]. Copyright 2013 American Chemical Society. BHQ, black hole fluorescence quencher.

In addition, different kinds of dye molecules can be capsulated inside one single SiNP simultaneously to construct large Stokes shifting fluorescence probes based on the principle of FRET, the ratiometric fluorescence platform and the fluorescence “turn on” nanosensor. A ratiometric pH nanoprobe was based on two-chromophore-doped SiNPs [31]. These two dye-doped novel SiNPs were synthesized via the microemulsion method. FITC was used as a pH-sensitive indicator, while RuBpy as a reference dye. The system can noninvasively monitor the changes of intracellular pH. This novel pH nanosensor could monitor changes of intracellular pH. In addition, the silica shell can protect the dyes from direct environmental effects, so lengthening the lifetime in a cell and making the nanosensors highly reversible and sensitive. As another example, Ju’s group designed an MSN with fluorescein and black hole fluorescence quencher (BHQ) inside and realized *in situ* “turn-on” imaging of telomerase activity *in vivo* [76]. BHQ, immobilized on the inner pore surface of MSNs through a covalently bond, could quench the fluorescence of loaded fluorescein. The gatekeeper, wrapping DNA, can be extended when the telomerase and deoxynucleotide dNTPs existed. The extended DNA is designed to form a hairpin-like DNA structure and to move away from the MSNs. The moving of the gatekeeper released fluorescein for “turn-on” fluorescent imaging of telomerase activity at the live cell level. The whole experiment design is shown in Figure 10.

Wang *et al.* also presented FRET-mediated NIR fluorescent dye molecule-doped fluorescent SiNPs, as we introduced above [30]. Moreover, different from being synthesized by reverse microemulsion, two types of perylenediimide (PDI) derivatives were covalently incorporated in one single SiNP by the Stöber method [77]. Tan and co-workers reported the triple-dye-doped FRET SiNPs [78]. They chose reference dye 6-carboxy-X-rhodamine (ROX), 5-carboxyrhodamine 6G (R6G) and FITC in their experiment construction for their effective fluorescence spectral overlapping. In this design, FITC was selected as a donor for both R6G and ROX. R6G was used as a donor for ROX and an acceptor for FITC at the same time. By changing the doping ratio of the three organic fluorescent dyes, the FRET-mediated emission signal can be simply tuned, and the SiNPs displayed different colors under only one single wavelength excitation. In this way, under a short-wavelength excitation, the longest-wavelength fluorescent emission spectrum will be exhibited. With the large Stokes shift, these FRET SiNPs can be detected in samples with Rayleigh/Raman scattering. Moreover, they employed the avidin-biotin binding experiment as a model to ensure the further application with biomolecules, such as aptamers and proteins. This demonstrated that biotin-modified SiNPs successfully covered the streptavidin-coated microspheres. Recently, multiple NIR dye-doped SiNPs as imaging agents for different experimental disease models have also been presented [79]. With single dye doping, the loading inside the silica matrix is always low, and the self-quenching phenomena often occur. When two different NIR dyes existed, the aggregation probability among the same variety of dye decreased, which could increase the SiNPs’ doping efficiency and decrease the self-quenching processes. After studying the whole-body and *ex vivo* NIR fluorescence to investigate the distribution, they showed that FRET SiNP shave an application as a contrast for *in vivo* tumor imaging and cell tracking.

## 4. Challenges and Perspective

Fluorescent imaging is a widely-used tool in biological research. The ever-increasing demand for monitoring multiple biological processes simultaneously calls for fluorescent probes that can meet many requirements, including, but not limited to, high stability and sensitivity in complex environments, good solubility and a proper emission spectrum. The unique characteristics, excellent biocompatibility, non-toxicity, high hydrophily, optically transparent, size-tunable and ease to covalently attach to multifarious biomolecules, have enabled SiNPs to construct fluorescent probes for effective imaging inside living cell and at the small animal level. Due to the robust and bright emitting light, fluorescent SiNPs have emerged as potential ﬂuorescent labels. Although enormous advances have been made using SiNPs in labeling, separation, bioimaging, disease diagnosis and therapy, several limitations hinder their further advancement. Firstly, the leakage of dyes from the fluorescent SiNPs over time, which could significantly shrink the fluorescence intensity of the particle and increase the background signal, needs further investigation. Secondly, the nonspecific binding of SiNPs needs to be settled. Thirdly, it is vital to standardize the procedure of the synthesis and functionalization to guarantee that SiNPs have a controllable size, good dispensability in aqueous solution and uniform multifunction. Moreover, to achieve ultrasensitive analysis, many fluorescent dye molecules need to be co-encapsulating inside one single SiNP. The multifunctional fluorescent SiNPs with the capabilities of diagnosis and therapeutics attract more attention, since they have great potential for providing an attractive see-and-treat strategy to deal with tumors or other diseases. However, SiNPs still have a very long way to translate the functionalized formulation systems into the medical market. We expect that SiNP-based nanoparticles can make significant progress and be applied in various clinical applications in the future.
